# iNOS/NOX-mediated stress, telomerase activity, and hTERT expression as potential biomarkers in sleep apnea

**DOI:** 10.1007/s11845-026-04334-x

**Published:** 2026-04-06

**Authors:** Nuray Üremiş, Deniz Tuncel Berktaş, Fatma İnanç Tolun, Muhammed Mehdi Üremiş

**Affiliations:** 1 Department of Medical Biochemistry, Medical Faculty, Kahramanmaraş Sütçü İmam University, Kahramanmaraş, Turkey; 2 Department of Neurology, Medical Faculty, Kahramanmaraş Sütçü İmam University, Kahramanmaraş, Turkey

**Keywords:** Nitrosative stress, Obstructive sleep apnea, Oxidative stress, Redox balance, Telomere length, Thiol/disulfide homeostasis

## Abstract

**Background:**

Obstructive sleep apnea (OSA) is a sleep disorder caused by recurrent upper airway obstruction during sleep.

**Aim:**

This study aims to evaluate NADPH oxidase (NOX)/inducible nitric oxide synthase (iNOS)-mediated stress biomarkers in sleep apnea patients. In addition, human telomerase reverse transcriptase (hTERT) expression and telomerase activity were analyzed to elucidate the relationship between sleep apnea and cellular aging processes.

**Methods:**

The study included 47 OSA patients and 45 healthy control subjects. Telomerase enzyme activity and hTERT levels were measured to evaluate the relationship between OSA and cellular senescence mechanisms. Oxidative/nitrosative stress was analysed by measuring total oxidant/antioxidant status (TOS/TAS), oxidative stress index (OSI), NOX, total and native thiol, disulfide, iNOS, nitric oxide (NO), and 3-nitrotyrosine (3-NT)**.**

**Results:**

A significant increase in NOX, TOS, OSI, iNOS, 3-NT, NO, and a substantial decrease in SOD, TAS, total thiol, native thiol, and disulfide levels were found in the serum of OSA patients. A significant decrease was detected in both telomerase activity (*p* < 0.05) and hTERT levels (*p* < 0.001) in OSA patients, and the reduction in hTERT levels was statistically more pronounced. A positive correlation was found between the apnea-hypopnoea index and iNOS. hTERT level showed high discriminative performance in diagnosing OSA with an AUC value of 0.875.

**Conclusions:**

Decreased hTERT-mediated cellular responses and increased oxidative/nitrosative modifications at the macromolecular level are among the basic mechanisms of OSA pathogenesis.

## Introduction

Obstructive sleep apnea (OSA) is a disease in which sleep quality is impaired by apnea and hypopnea episodes resulting from recurrent obstruction of the upper airway during sleep [1]. It is estimated that approximately 936 million adults aged 30–69 years worldwide have mild-to-severe OSA, while nearly 425 million are affected by moderate-to-severe disease [2]. The risk of OSA increases markedly with advancing age, obesity, and recurrent hypoxia–reoxygenation cycles [3]. In OSA, loss of tonus of the muscles in the pharyngeal region causes partial or complete airway closure [4]. The resulting intermittent cycles of hypoxia and reoxygenation may be associated with increased iNOS (inducible nitric oxide synthase) expression, NO, reactive oxygen species (ROS), and reactive nitrogen species (RNS) production [5]. iNOS-mediated 3-NT is a marker of NO reaction with superoxide radicals, protein nitration and oxidative damage [6]. Elevated iNOS levels affect vascular function by contributing to oxidative stress and inflammation in OSA patients [5, 7]. Dysregulation of NO production and bioavailability in OSA impairs vascular function and contributes to endothelial dysfunction [8]. NOX is an enzyme complex that produces ROS in various cells and contributes to oxidative stress [6]. The balance between NO and ROS, including superoxide produced by NOX, is crucial for maintaining vascular homeostasis in sleep apnea [9].

Telomerase is an enzyme that prolongs the replicative lifespan of cells, and this activity is mediated by the catalytic subunit of the enzyme human telomerase reverse transcriptase (hTERT) [10]. Studies have suggested a potential link between oxidative stress, inflammation, telomere shortening, and telomerase activity in various diseases, including those associated with sleep apnea [11, 12]. TERT gene variants and oxidative stress dynamics in OSA may affect telomerase function, potentially contributing to cellular dysfunction and aging processes [13, 14].

The aims of examining nitrosative stress markers such as iNOS, NOX, 3-NT, NO, associated with oxidative stress parameters such as TAS, TOS, OSI, total thiol, native thiol, SOD in sleep apnea patients are multifaceted and can provide valuable information about the pathophysiology, complications and potential therapeutic targets associated with the disease [15]. Studies have shown that decreased antioxidant and increased oxidant levels are associated with OSA [16, 17]. However, the relationship between changes in telomerase activity and hTERT expression and cellular damage and ageing processes in this disease has not been comprehensively addressed [12]. In this context, our study aims to reveal the association of iNOS and NOX-mediated oxidative/nitrosative stress biomarkers, telomerase activity, and hTERT expression levels with sleep apnea and the molecular interactions between these parameters. Revealing the complex mechanisms involving oxidative/nitrosative stress and telomerase interaction in individuals with sleep-disordered breathing will facilitate the development of targeted interventions.

## Patients and methods

### Study population

This study was conducted with the participation of individuals diagnosed with obstructive sleep apnea who were admitted to Kahramanmaraş Sütçü İmam University (KSU) Research Hospital Neurology Outpatient Clinic and/or hospitalized in the Neurology Service. The research was carried out with the ethical approval of the KSU Medical Research Ethics Committee with protocol number 2024/18–151. The patient group comprised 47 adults aged 18–65 with an apnea-hypopnoea index (AHI) ≥ 5. Stop-Bang questionnaire was applied to determine the risk level for OSA. Total scores ranged from 0 to 8, and a score of ≥ 3 was considered high risk for OSA. In the control group, 45 healthy individuals without neurological or chronic disease or regular medication use were included.

### Sleep study and polysomnography scoring

Nighttime sleep was evaluated using an Embla® S7000 PSG amplifier (Flaga, Reykjavik, Iceland). A total of 21 signals, such as respiratory signals, EEG, ECG, EOG, EMG, snoring, and patient position, were recorded on the PSG unit. Polysomnography was scored according to The AASM Manual for the Scoring of Sleep and Associated Events Rules, Terminology, and Technical Specifications Version 3. Obstructive hypopnea was defined as a reduction of at least 30% in airflow lasting at least 10 s and a fall of 3% in SaO_2_ or accompanying arousal. Apnea is defined as a reduction of at least 90% airflow amplitude and respiratory issues lasting at least 10 s. According to the International Classification of Sleep Disorders (ICSD-3 TR), OSA is diagnosed when there is an AHI > 5 according to the PSG report and snoring, witnessed apnea, and one or more of the daytime sleepiness diagnostic criteria. According to the AHI, the severity of OSA is evaluated as mild at AHI 5–14, moderate at AHI 15–29, and severe at AHI 30 and above. We included only patients with an AHI of 30 or above in our study. None had yet started PAP treatment.

### Measurement of serum hTERT, telomerase, NOS2/iNOS, NOX and 3-NT levels

All blood samples obtained from the patient and control groups were collected in biochemistry tubes containing gel. Serum fractions were separated by centrifugation at 4000 rpm for 7 min and stored in a −80 °C freezer until the analysis day. Serum hTERT, telomerase, NOS2/iNOS, NOX, and 3-NT levels were quantitatively determined using commercial ELISA kits (Elabscience; EL-H0164, EL-H0706, EL-H0753, EL-H2223, EL-0040) according to the manufacturer’s instructions. Briefly, standards and serum samples were loaded onto antibody-coated microtiter plates. Biotin-conjugated secondary antibody and streptavidin-HRP solutions were then added and incubated, respectively. After washing, color development was achieved by adding the substrate solution, and absorbance values were measured spectrophotometrically at 450 nm wavelength. Concentrations were calculated using standard curves, and results were expressed in ng/mL or pg/mL.

### Colorimetric measurement of serum NO and SOD levels

A colorimetric commercial kit (Elabscience E-BC-K-135-M) determined serum NO level. The measurement procedure consists of two main stages: The incubation stage and the chromogenic reaction stage. In the incubation step, 30 µL of substrate and 30 µL of enzyme solution were added per well to prepare the enzyme working mixture. To prepare the chromogenic working mixture, 50 µL chromogenic A solution, 20 µL chromogenic B solution, and 20 µL acid reagent were added to each well, and the mixture was homogenized. As part of the study procedure, 80–100 µL serum sample was taken into an eppendorf tube, followed by the addition of 60 µL enzyme working mixture. After incubating the mixture at 37 °C for 60 min, 20 µL sulfate solution and 10 µL alkaline reagent were added. Finally, the mixture was centrifuged at 10,000 g for 10 min, and the supernatant was used for analysis. In the chromogenic reaction step, 50 µL of the chromogenic working solution was added to each well of a 96-well microplate. Standards and samples were pipetted into the respective wells in a volume of 120 µL, and the mixture was incubated for 5 min at room temperature. After the reaction, absorbance values were measured in a microplate reader at 530 nm wavelength. The method used for SOD measurement is based on the principle of measuring the blue formazan complex formed by the reduction of nitroblue tetrazolium (NBT) by superoxide radicals produced by xanthine oxidase [18, 19].

### Determination of serum TOS, TAS, OSI, total thiol, native thiol and disulfide levels

Serum total antioxidant (TAS), oxidative stress index (OSI), total oxidant levels (TOS), and thiol levels were measured using colorimetric kits (Rel Assay Diagnostics). Analyses were performed using the procedures described previously in detail [20–23]. TAS values were calculated using the $\frac{\Delta Ab{s}_{{H}_{2}O} - \Delta Ab{s}_{Sample} }{\Delta Ab{s}_{{H}_{2}O} - \Delta Ab{s}_{Standart}}$ formula based on the procedures performed according to the TAS Kit procedure. Similarly, TOS values were calculated using the $\frac{\Delta Ab{s}_{Sample}}{\Delta Ab{s}_{Standart}}x10$ formula after the procedures were performed according to the TOS kit procedure. OSI was determined by the ratio of TOS levels to TAS levels (TOS/TAS). In the determination of total thiol, Reagent 1 and then Reagent 2 were added to the samples according to the kit protocol, and then the first absorbance was measured at 700/415 nm wavelengths. Then, the second absorbance was measured with the addition of Reagent 3, and the difference between these values represents SH + SS. The determination of native thiol (SH) was based on the difference between the first absorbance obtained by adding Reagent 1 to the sample and the second after adding Reagent 2 by the kit procedure. The dynamic disulfide level was calculated using the native and total thiol values obtained (SH/[SH + SS] ratio).

### Statistical analysis

All statistical analyses were performed using GraphPad Prism software (version 10.0 and above). An independent samples t-test was applied to compare telomerase dynamics and oxidative/nitrosative stress parameters between control and OSA (obstructive sleep apnea) groups. The relationship between telomerase activity, redox balance, and biomarkers related to serum thiol/disulfide homeostasis in sleep apnea patients was evaluated by Spearman or Pearson correlation analyses. p < 0.05 was accepted as the limit of statistical significance. In addition, receiver operating characteristic (ROC) curve analysis and area under the curve (AUC) calculations were performed to determine the diagnostic power of hTERT, telomerase, NO, NOX, 3-NT, TAS, TOS, OSI, and iNOS levels.

## Results

### Clinical characteristics

The comparative evaluation of demographic and clinical parameters of individuals diagnosed with OSA and the healthy control group is presented in Table 1. The mean age of the individuals in the OSA group was 49.6 ± 9.3 years, while the mean age in the control group was 39.6 ± 10.4 years. When the gender distribution of the OSA group was analyzed, it was observed that the proportion of male individuals was significantly higher. When the diagnostic and evaluation criteria of the OSA group were analyzed, the mean Stop-Bang score of the patients was 5.15 ± 1.3, suggesting that the participants had a high-risk profile for OSA. The AHI values evaluated by polysomnography were determined as 66.0 ± 23.8, and this result revealed that most individuals were in the severe OSA group. In addition, the mean minimum oxygen saturation was 75 ± 11.1%, indicating that apnea and hypopnoea episodes caused significant hypoxemia in the patients. Taken together, these findings suggest that both subjective screening (Stop-Bang) and objective assessment (AHI and oxygen saturation) parameters are compatible with the severity of the disease in individuals diagnosed with OSA.

**Table 1:** Demographic characteristics of the study groups and diagnostic and evaluation criteria of the obstructive sleep apnea (OSA) group

	Control		OSA	
	Range	Mean ± SD	Range	Mean ± SD
*n* Gender (M/F)	45 25/20		47 42/5	
Age	19 - 67	39.6 ± 10.4	27 −68	49.6 ± 9.3
Stop-Bang Score	0–2	1,1 ± 0,6	3 - 8	5.15 ± 1.3
AHI (Apnea–Hypopnea Index)	-	-	31 - 117	66.0 ± 23.8
Minimum Oxygen (%)	-	-	50 - 89	75 ± 11.1

### Serum telomerase dynamics, oxidative/nitrosative stress, and redox homeostasis biomarker levels

**Fig. 1:**
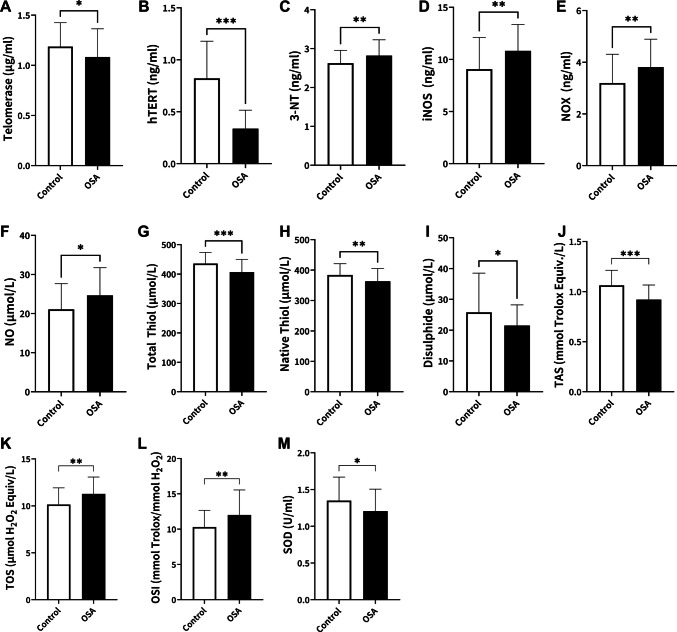
Comparison of Telomerase (**A**), hTERT (**B**), 3-NT (**C**), iNOS (**D**), NOX (**E**), NO (**F**), Total Thiol (**G**), Native Thiol (**H**), Disulfide (**I**), TAS (**J**), TOS (**K**), OSI (**L**) and SOD (**M**) levels in the serum of OSA patients with a control group and patient groups. *p < 0.05, **p < 0.01, ***p < 0.001. Abbreviations: hTERT, human telomerase reverse transcriptase; NOX, NADPH Oxidase; iNOS, inducible nitric oxide synthase; NO, nitric oxide; 3-NT, 3-nitrotyrosine; DS, disulfide; NT, native thiol; TT, total thiol; OSI, oxidative stress index; TAS, total antioxidant, status; TOS, total oxidant status; SOD, superoxide dismutase

The serum levels of the telomerase complex, oxidative/nitrosative stress, and redox homeostasis biomarkers in individuals with OSA and the healthy control group are presented in Fig. 1 and Table 2. In the OSA group, significant decreases were found in both total telomerase activity and hTERT levels compared to the control group (*p* < 0.05 and *p* < 0.001, respectively); especially, the decrease in hTERT levels was more pronounced. This result suggests that repeated episodes of hypoxia-reoxygenation inhibit the adequate synthesis of the telomerase enzyme complex that elongates telomere DNA in the cell nucleus and accelerates cellular senescence processes. Nitrosative stress indicators 3-nitrotyrosine (3-NT), iNOS, and NOX levels were significantly increased in OSA patients (*p* < 0.01, *p* < 0.01, and *p* < 0.05). In addition, peripheral NO concentration was significantly increased in the apnea group compared to the control group (*p* < 0.05). The NOX/iNOS increase and 3 NT accumulation observed in OSA patients indicate that excessive production of reactive oxygen and nitrogen species caused by recurrent hypoxia-reoxygenation periods triggers nitroxidative damage in cellular components. In antioxidant defense mechanisms, SOD, TAS, total thiol, and native thiol levels were significantly decreased in the OSA group, whereas the oxidative stress indicator TOS and the oxidant/antioxidant balance indicator OSI were increased (decrease in TAS and disulfide *p* < 0.001 and *p* < 0.01; increase in TOS and OSI *p* < 0.01). A low but significant reduction in disulfide levels was observed in the OSA group (*p* < 0.05). These results suggest that OSA weakens the thiol-based redox buffer system and disrupts the oxidant/antioxidant balance.

**Table 2:** Biochemical parameters of OSA and healthy control group

	Control (Mean ± SD)	OSA (Mean ± SD)	*p-v*alue Control vs. OSA
hTERT	0.82 ± 0.36	0.34 ± 0.18	< 0.001
Telomerase	1.2 ± 0.24	1.1 ± 0.28	0.05
3-NT	2.6 ± 0.33	2.8 ± 0.40	0.009
iNOS	9.1 ± 3.1	11 ± 2.5	0.002
NOX	3.2 ± 1.1	3.8 ± 1.1	0.006
NO	21 ± 6.6	25 ± 7.1	0.01
Native Thiol	384 ± 37	364 ± 41	0.01
Total Thiol	436 ± 37.7	407 ± 43.7	< 0.001
TAS	1.06 ± 0.15	0.92 ± 0.146	< 0.001
TOS	10.1 ± 1.77	11.3 ± 1.80	0.002
SOD	1.35 ± 0.321	1.21 ± 0.299	0.02
OSI	10.3 ± 2.38	12 ± 3.54	0.004

### Correlation of telomerase dynamics, oxidative/nitrosative stress, and redox homeostasis biomarkers

**Fig. 2:**
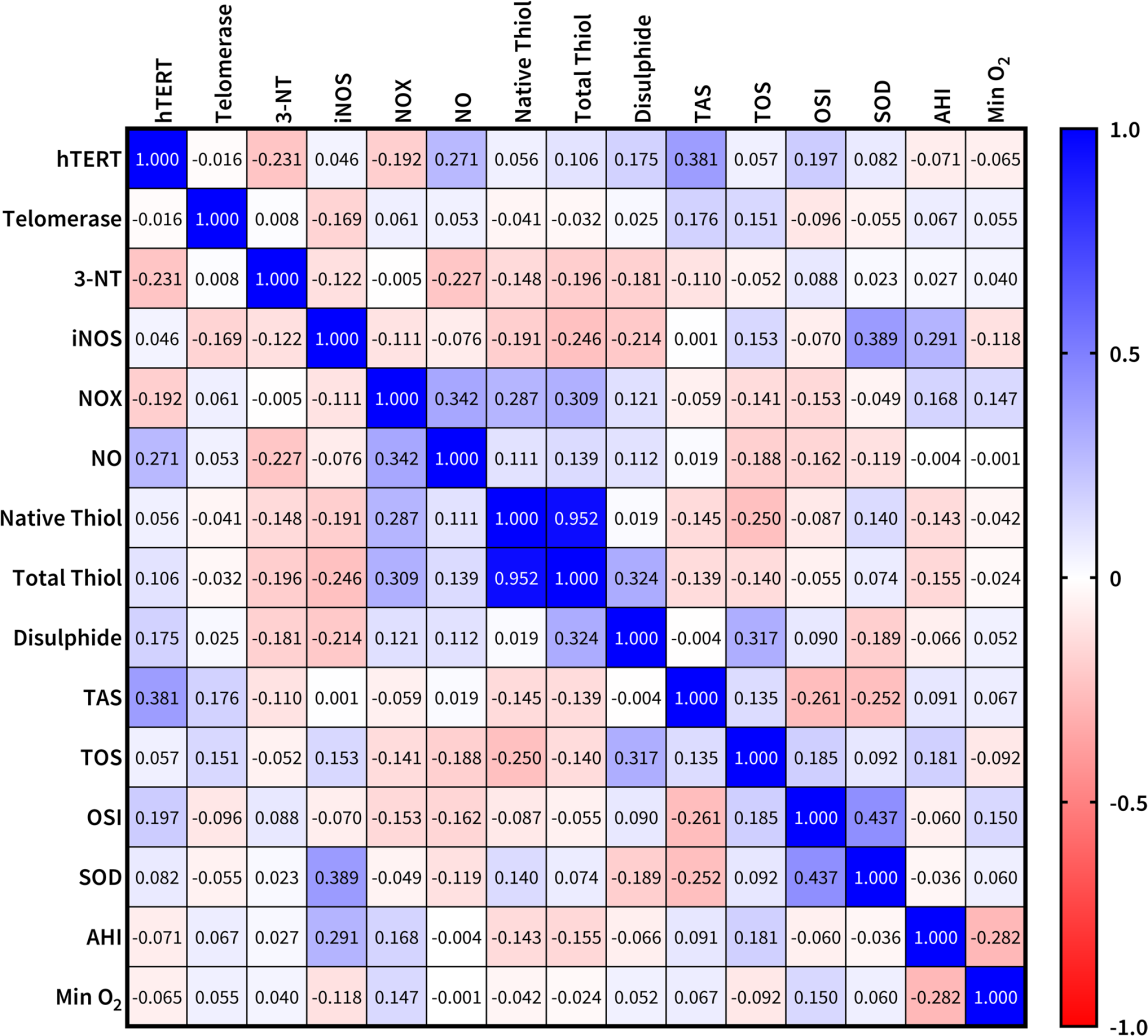
Heatmap of Spearman Correlation Coefficients in OSA Patients. This heatmap visualizes the correlation coefficients (r) from Table 2. The color scale (shown on the right) ranges from −1.0 (strong negative correlation) to + 1.0 (strong positive correlation). Shades of red represent negative correlations, whereas shades of blue represent positive correlations. The intensity of the color corresponds to the magnitude of the correlation, allowing for a quick visual assessment of the relationships among the measured parameters

Significant findings were obtained in correlation analyses to evaluate the relationships between telomerase, hTERT, and oxidative/nitrosative stress-related biomarkers in serum of patients with OSA (Fig. 2, Table 3). A significant positive correlation was found between hTERT levels and TAS (r = 0.381, *p* = 0.008). This suggests that increased hTERT levels may be related to systemic antioxidant capacity. In addition, significant positive correlations were also observed between NOX levels and native thiol (r = 0.287, *p* = 0.050) and total thiol (r = 0.309, *p* = 0.035) levels. The positive correlation between total thiol and disulfide levels was also statistically significant (r = 0.324, *p* = 0.026), supporting the relationship between thiol/disulfide balance and oxidative stress. A significant positive correlation was also found between disulfide levels and TOS (r = 0.317, *p* = 0.030). However, a strong positive correlation was found between OSI and SOD activity (r = 0.437, *p* = 0.002), suggesting that increased oxidative stress may stimulate the defense system. Likewise, there was a significant positive correlation between iNOS and SOD (r = 0.389, *p* = 0.007), suggesting a possible balance mechanism between nitrosative stress and antioxidant response. These findings indicate that the relationships between telomerase activity, thiol/disulfide homeostasis, and oxidative and nitrosative stress indicators may play a decisive role in elucidating the biochemical mechanisms of OSA.

**Table 3:** Evaluation of the correlation of telomerase, hTERT and oxidative/nitrosative stress-related biomarkers in obstructive sleep apnea patients

		hTERT	Telomerase	3-NT	iNOS	NOX	NO	Native thiol	Total thiol	Disulphide	TAS	TOS	OSI	SOD
hTERT	*r*	1000	−0.016	−0.231	0.046	−0.192	0.271	0.056	0.106	0.175	0.381	0.057	0.197	0.082
	p		0.916	0.118	0.761	0.197	0.065	0.710	0.476	0.239	0.008	0.702	0.184	0.584
Telomerase	*r*	−0.016	1.000	0.008	−0.169	0.061	0.053	−0.041	−0.032	0.025	0.176	0.151	−0.096	−0.055
	*p*	0.916		0.956	0.255	0.684	0.723	0.783	0.833	0.870	0.238	0.312	0.523	0.715
3-NT	*r*	−0.231	0.008	1.000	−0.122	−0.005	−0.227	−0.148	−0.196	−0.181	−0.110	−0.052	0.088	0.023
	*p*	0.118	0.956		0.412	0.973	0.124	0.321	0.188	0.223	0.462	0.727	0.558	0.876
iNOS	*r*	0.046	−0.169	−0.122	1.000	−0.111	−0.076	−0.191	−0.246	−0.214	0.001	0.153	−0.070	0.389
	*p*	0.761	0.255	0.412		0.458	0.610	0.199	0.095	0.148	0.993	0.306	0.642	0.007
NOX	*r*	−0.192	0.061	−0.005	−0.111	1.000	0.342	0.287	0.309	0.121	−0.059	−0.141	−0.153	−0.049
	*p*	0.197	0.684	0.973	0.458		0.018	0.050	0.035	0.416	0.693	0.344	0.305	0.745
NO	*r*	0.271	0.053	−0.227	−0.076	0.342	1.000	0.111	0.139	0.112	0.019	−0.188	−0.162	−0.119
	*p*	0.065	0.723	0.124	0.610	0.018		0.460	0.352	0.452	0.899	0.205	0.275	0.424
Native Thiol	*r*	0.056	−0.041	−0.148	−0.191	0.287	0.111	1.000	0.952	0.019	−0.145	−0.250	−0.087	−0.119
	p	0.710	0.783	0.321	0.199	0.050	0.460		0.000	0.899	0.331	0.090	0.561	0.349
Total Thiol	*r*	0.106	−0.032	−0.196	−0.246	0.309	0.139	0.952	1.000	0.324	−0.139	−0.140	−0.055	0.074
	*p*	0.476	0.833	0.188	0.095	0.035	0.352	0.000		0.026	0.353	0.349	0.714	0.620
Disulphide	*r*	0.175	0.025	−0.181	−0.214	0.121	0.112	0.019	0.324	1.000	−0.004	0.317	0.090	−0.189
	*p*	0.239	0.870	0.223	0.148	0.416	0.452	0.899	0.026		0.978	0.030	0.549	0.202
TAS	*r*	0.381	0.176	−0.110	0.001	−0.059	0.019	−0.145	−0.139	−0.004	1.000	0.135	−0.261	−0.252
	*p*	0.008	0.238	0.462	0.993	0.693	0.899	0.331	0.353	0.978		0.364	0.077	0.087
TOS	*r*	0.057	0.151	−0.052	0.153	−0.141	−0.188	−0.250	−0.140	0.317	0.135	1.000	0.185	−0.252
	*p*	0.702	0.312	0.727	0.306	0.344	0.205	0.090	0.349	0.030	0.364		0.213	0.539
OSI	*r*	0.197	−0.096	0.088	−0.070	−0.153	−0.162	−0.087	−0.055	0.090	−0.261	0.185	1.000	0.437
	*p*	0.184	0.523	0.558	0.642	0.305	0.275	0.561	0.714	0.549	0.077	0.213		0.002
SOD	*r*	0.082	−0.055	0.023	0.389	−0.049	−0.119	0.140	0.074	−0.189	−0.252	0.092	0.437	1.000
	*p*	0.584	0.715	0.876	0.007	0.745	0.424	0.349	0.620	0.202	0.087	0.539	0.002	

Abbreviations: *hTERT* human telomerase reverse transcriptase, *OSI* oxidative stress index, *NOX* NADPH oxidase, inducible nitric oxide synthase, *3-NT* 3-nitrotyrosine, *NO* nitric oxide, *TAS* total antioxidant status, *TOS* total oxidant status, *SOD* superoxide dismutase

### Correlation analysis between apnea-hypopnoea index and biochemical parameters

In the study, the relationship between AHI and various biochemical parameters in OSA patients was investigated, and the results of correlation analysis are presented in Table 4 and Fig. 2. As a result, the study found a statistically significant and positive correlation between AHI and iNOS levels (r = 0.291, *p* = 0.047), suggesting that increased apnea-hypopnoea severity may be associated with increased iNOS expression. No significant correlation was found between AHI and other parameters such as hTERT, telomerase, 3-NT, NOX, NO, NO, thiol/disulfide balance components, TAS, TOS, OSI, and SOD levels (*p* > 0.05). These findings support the role of nitric oxide-derived mediators in the pathophysiology of OSA and show that iNOS is associated with disease severity.

**Table 4:** Correlation analysis between apnea–hypopnea index and biochemical parameters in OSA patients

Parameters	Test value	AHI
hTERT	r	−0.071
	*p*	0.634
Telomerase	r	0.067
	*p*	0.656
3-NT	r	0.027
	*p*	0.856
iNOS	r	0.291
	*p*	0.047
NOX	r	0.168
	*p*	0.258
NO	r	−0.004
	*p*	0.980
Native Thiol	r	−0.143
	*p*	0.339
Total Thiol	r	−0.155
	*p*	0.298
Disulphide	r	−0.066
	*p*	0.661
TAS	r	0.091
	*p*	0.542
TOS	r	0.181
	*p*	0.223
OSI	r	−0.060
	*p*	0.691
SOD	r	−0.036
	*p*	0.812

### Correlation analysis between minimum oxygen saturation and biochemical parameters

In this study, the results of the correlation analysis between minimum oxygen saturation (Min O₂) and various biochemical parameters in OSA patients are presented in Table 5 and Fig. 2. The correlation coefficients for the analysed parameters were low, and no statistical significance was observed (p > 0.05). These findings indicate that changes in minimum oxygen levels in the individuals in our study did not have a significant effect on telomerase activity, oxidative stress markers, and the antioxidant defence system.

**Table 5:** Correlation analysis of the biochemical parameters of minimum oxygen

Parameters	Test value	Min O_2_
hTERT	r	−0.065
	*p*	0.664
Telomerase	r	0.055
	*p*	0.711
3-NT	r	0.040
	*p*	0.792
iNOS	r	−0.118
	*p*	0.428
NOX	r	0.147
	*p*	0.325
NO	r	−0.001
	*p*	0.994
Native Thiol	r	−0.042
	*p*	0.778
Total Thiol	r	−0.024
	*p*	0.873
Disulphide	r	0.052
	*p*	0.727
TAS	r	0.067
	*p*	0.653
TOS	r	−0.092
	*p*	0.540
OSI	r	0.150
	*p*	0.314
SOD	r	0.060
	*p*	0.690

### Diagnostic values of SOD, TOS, TAS, OSI, total thiol, native thiol, disulfide, 3-NT, iNOS, NOX, hTERT and telomerase

**Fig. 3:**
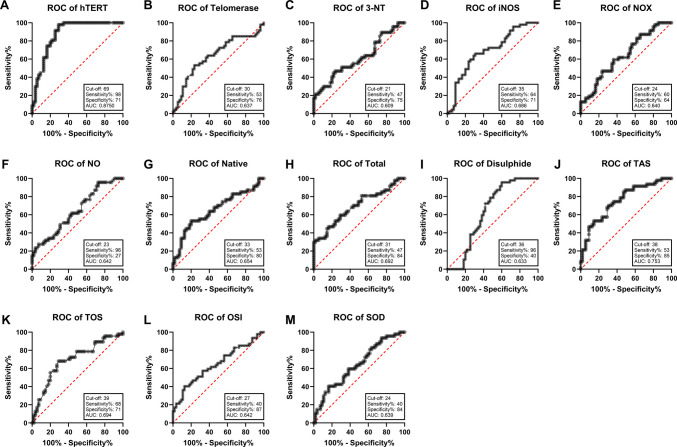
ROC analyses of hTERT (**A**), Telomerase (**B**), 3-NT (**C**), iNOS (**D**), NOX (**E**), NO (**F**), Native Thiol (**G**), Total Thiol (**H**), Disulfide (**I**), TAS (**J**), TOS (**K**), OSI (**L**), and SOD (**M**) biomarkers in OSA patients. Graphs show cut-off values, the area under the curve (AUC), and sensitivity and specificity values

In our study, the diagnostic values of various oxidative stress markers, antioxidant defense system components, and parameters related to telomere metabolism evaluated in OSA patients were analyzed by ROC curves (Fig. 3). The AUC value calculated for hTERT was 0.875 and determined as the parameter with the highest diagnostic accuracy with 98% sensitivity and 71% specificity. hTERT is a strong candidate for use as a biomarker in diagnosing OSA.

When the diagnostic performance of TAS and TOS levels were analyzed, it was observed that they had 53% and 68% sensitivity, 85% and 71% specificity, and AUC values of 0.753 and 0.694, respectively. Among the parameters evaluated in the study, hTERT, TAS, and TOS markers with the highest diagnostic accuracy were followed by Total thiol, iNOS, Native thiol, NO, OSI, NOX, and SOD, respectively. The AUC values of these parameters ranged between 0.692 and 0.639. Telomerase and 3-NT levels were also found to have a certain discriminative power. The significant diagnostic performances of hTERT related to cell aging, parameters related to oxidative/nitrosative stress (TAS, TOS, iNOS, NOX), and thiols related to antioxidant defense support that cellular redox balance and genomic stability are impaired in the pathophysiology of OSA.

## Discussion

This study aimed to evaluate the endogenous antioxidant-oxidant balance by examining NOX/iNOS-mediated oxidative and nitrosative stress levels and thiol/disulfide homeostasis in OSA patients. It also reveals the relationship between telomerase-based aging mechanisms and the pathophysiological processes of OSA. In the study, disulfide, total thiol, and native thiol levels were examined to reveal the physiopathological role of serum thiol pool; NOX, TAS, TOS, OSI, SOD, iNOS, NO, and 3-NT markers were evaluated to reflect net oxidative stress levels and redox homeostasis; hTERT and telomerase levels were determined to assess cellular senescence mechanisms.

Hypoxia and reoxygenation conditions lead to high ROS and NO production levels due to the induction of NOX and iNOS enzymes [24]. This process is followed by the accumulation of hydrogen peroxide and superoxide in the cell and an increase in S-nitrosated compounds [6, 25]. ROS and RNS, which have high reactivity on proteins, lipids, and nucleic acids, disrupt the structure and function of cellular components by regulating oxidative and nitrosative modifications [6]. Thiol-containing endogenous compounds provide an antioxidant defence mechanism by neutralising these oxidant and nitrosating compounds [26]. In studies, increased ROS and RNS levels and decreased antioxidant defense capacity have been closely associated with the pathogenesis of OSA. In obstructive sleep apnea (OSA) patients treated with continuous positive airway pressure (CPAP) therapy, changes in serum thiol-disulfide balance before and after treatment were investigated; it was reported that thiol levels, which were low before therapy in individuals with moderate and severe OSA, increased significantly after CPAP treatment [26]. In a study involving a large group of OSA patients (*n* = 230), total and native thiol levels and disulfide levels were found to be low about the patient’s apnea–hypopnea index [27]. In the study evaluating the contribution of endothelial dysfunction and oxidative stress in OSA, TAS, TOS, OSI, and NO levels were examined in OSA patients who had not received pharmacological treatment in the last 15 days. While a significant increase was observed in TOS and OSI levels in the OSA group, no significant difference was found in TAS and NO levels [28]. In OSA patients with comorbidity, TOS levels increased significantly in parallel with the increase in AHI and comorbidity severity, whereas TAS and oxygen desaturation levels decreased significantly [17]. In the study in which oxidative stress status in the uvular mucosa of individuals with OSA was evaluated, it was reported that TOS and OSI increased and TAS decreased in mild, moderate and severe OSA groups [29]. Although there are studies on various biomarkers related to oxidative and nitrosative stress in OSA patients, data on iNOS and NOX enzymes are limited. In an evaluation of patients with and without OSA after lung transplantation, it was reported that iNOS levels increased in the OSA group in correlation with AHI [5]. It was shown that iNOS, COX-2, and nitrotyrosine immunofluorescence signal intensities decreased in venous endothelial cell samples obtained from obstructive sleep apnea (OSA) patients before and after CPAP treatment [7]. A-930G and C242T polymorphisms of the NOX complex were investigated in OSA patients; A-930G polymorphism was found to be prominent in terms of genetic predisposition [30]. Unlike previous studies, NOX, which is the primary enzymatic source of vascular oxidative stress, NOX-related oxidant biomarker levels, and thiol/disulfide homeostasis were investigated in this study. However, modulators of the iNOS/NOX/3-NT-mediated oxidative-nitrosative stress pathway, which are thought to play a role in the pathogenesis of OSA, were associated with the disease in this study for the first time in the literature. In this study, NOX, iNOS, 3-NT, NO, TOS, and OSI levels were found to be significantly higher, and TAS, SOD, total thiol, and native thiol levels were found to be considerably lower in OSA patients compared to the control group. The results suggest that the systemic effects of OSA may lead to significant impairments in parameters such as the iNOS/NOX/3-NT axis and thiol/oxidative balance system at the serum level. In addition, high iNOS and NO levels in OSA may regulate vascular tone, blood flow, and inflammation.

Several studies have been carried out on oxidative stress parameters, nitric oxide, and thiol/disulfide balance to reveal the correlation of the mechanisms underlying the pathophysiology of OSA with the disease and its potential diagnostic value. The relationship between intermittent hypoxia and exhaled nitric oxide fraction (FeNO) was evaluated in OSA patients with comorbidity; however, no significant correlation was found between FeNO levels and AHI and obstructive apnea index (OAI) [31]. A study of 230 OSA patients found statistically significant correlations between total thiol, native thiol levels, and AHI. However, no significant correlation was found between disulfide, disulfide/native thiol, disulfide/total thiol, and native thiol/total thiol ratios and AHI [27]. Another study reported a negative correlation between TAS and AHI in OSA patients, whereas positive correlations were found between TOS and OSI and oxygen desaturation index [29]. Studies investigating the effects of CPAP therapy on oxidative stress in OSA have also been reported. In one meta-analysis, CPAP treatment was shown to significantly increase serum/plasma total antioxidant capacity (TAC) in patients with OSA, highlighting its potential regulatory role on oxidative stress [32]. In another study conducted in patients with severe OSA, short-term CPAP therapy was shown to partially reduce salivary oxidative stress markers (TBARS, AGEs, and AOPP) that increase during the night [33]. In addition, MDA (AUC: 0.854), which reflects lipid peroxidation and oxidative stress response, and antioxidant defense enzyme SOD (AUC: 0.750) have been reported to have significant discriminatory power in the diagnosis of OSA [34]. Although various studies in the literature reveal the correlations and diagnostic values ​​between OSA and oxidative stress-related biomarkers, no study has been found that examines the relationship between nitrosative stress biomarkers, NADPH oxidase, telomerase, and hTERT levels, and OSA and includes ROC analyses for these parameters. In this context, our study is the first to demonstrate a positive correlation between iNOS and OSA and that hTERT can be a strong diagnostic biomarker in OSA with an AUC value of 0.875.

Chronic intermittent hypoxia and reoxygenation cycles in OSA patients lead to DNA damage and telomere shortening via oxidative/nitrosative stress [35]. Telomere shortening is considered an important biomarker of biological aging [36]. In this context, a limited number of studies in the literature address the relationship between OSA and hTERT expression and telomerase activity [11, 13, 37]. A study examining the relationship between OSA and genetic polymorphisms related to the TERT gene reported that no significant relationship was found between disease severity and rs2853669 and rs2736100 polymorphisms [13]. In contrast, another study evaluating hypertensive OSA patients reported a negative association between the high apnea–hypopnea index and decreased telomerase activity in the blood [11]. A study evaluating telomerase and TER1 levels in newly diagnosed OSA patients and patients receiving CPAP treatment for 6 months showed that recurrent hypoxia reduces telomerase complex activity, and CPAP application improves this activity [37]. In a 6-month randomized, double-blind clinical trial, CPAP therapy was shown to attenuate leukocyte telomere shortening in patients with OSA, whereas a more pronounced telomere shortening was observed in the sham-CPAP group [38]. In another prospective study, intermittent hypoxia and sleep fragmentation associated with OSA were found to be related to telomere shortening, whereas a significant increase in telomere length was observed after 6 months of CPAP therapy [39]. These studies demonstrate that CPAP therapy may contribute to the preservation of telomere integrity. In this study, to better understand the dynamics of cellular aging in OSA patients, the telomerase enzyme levels and its catalytic subunit hTERT were examined, and the diagnostic values of these parameters were evaluated. The findings showed that both telomerase and hTERT levels decreased in the OSA group, and this decrease was especially pronounced in hTERT levels. In addition, ROC analysis revealed that hTERT has significant discriminatory power in diagnosing OSA.

## Conclusion

This study links the hTERT/telomerase system to OSA for the first time within the oxidative/nitrosative stress axis. It is well known in the literature that hypoxia accompanying OSA disrupts the oxidative balance. The findings we obtained in this study show that this disruption may cause both an increase in oxidative and nitrosative stress levels via the iNOS/NOX/3-NT pathway and an acceleration of cellular aging processes via the hTERT/telomerase pathway. Our study has three main findings: The first of these is that hTERT gene expression and telomerase activity are significantly low in OSA patients. The second important finding is that increased oxidative/nitrosative stress in patients suppresses thiol-based antioxidant defense mechanisms. Third, the change in hTERT and iNOS levels may be associated with OSA progression, and hTERT may be considered a biomarker for diagnostic or prognostic purposes. This study provides unique contributions to the literature. It paves the way for new research by demonstrating that cellular senescence and stress modulators are among the fundamental molecular mechanisms involved in the pathophysiology of OSA.

## Data Availability

The datasets generated during and/or analysed during the current study are available from the corresponding author on reasonable request.
